# Identification and Pathogenicity Evaluation of a Novel Reassortant Infectious Bursal Disease Virus (Genotype A2dB3)

**DOI:** 10.3390/v13091682

**Published:** 2021-08-25

**Authors:** Yulong Wang, Nan Jiang, Linjin Fan, Xinxin Niu, Wenying Zhang, Mengmeng Huang, Li Gao, Kai Li, Yulong Gao, Changjun Liu, Hongyu Cui, Aijing Liu, Qing Pan, Yanping Zhang, Xiaomei Wang, Xiaole Qi

**Affiliations:** 1 State Key Laboratory of Veterinary Biotechnology, Avian Immunosuppressive Diseases Division, Harbin Veterinary Research Institute, The Chinese Academy of Agricultural Sciences, Harbin 150069, China; xyylong@126.com (Y.W.); jiangnan3596@163.com (N.J.); 18346080676@163.com (L.F.); Nqct17@163.com (X.N.); unstoppable0402@outlook.com (W.Z.); huang.mm1017@foxmail.com (M.H.); gaoli@caas.cn (L.G.); likai@caas.cn (K.L.); gaoyulong@caas.cn (Y.G.); liuchangjun@caas.cn (C.L.); cuihongyu@caas.cn (H.C.); Liuaijing@caas.cn (A.L.); panqing@caas.cn (Q.P.); zhangyanping@caas.cn (Y.Z.); wangxiaomei@caas.cn (X.W.); 2 OIE Reference Laboratory for Infectious Bursal Disease, Harbin Veterinary Research Institute, The Chinese Academy of Agricultural Sciences, Harbin 150069, China; 3 Jiangsu Co-Innovation Centre for Prevention and Control of Important Animal Infectious Disease and Zoonosis, College of Veterinary Medicine, Yangzhou University, Yangzhou 225009, China

**Keywords:** infectious bursal disease virus, novel variant strain, genome, segment reassortment, pathogenicity

## Abstract

Infectious bursal disease virus (IBDV) is a non-enveloped, bi-segmented double-stranded RNA virus and the causative agent of a poultry immunosuppressive disease known as infectious bursal disease (IBD). The novel variant IBDV (nVarIBDV) recently posed a great threat to the development of the poultry industry. In this study, we identified a novel segment-reassortant IBDV strain, IBDV-JS19-14701 (Genotype A2dB3). Phylogenic analysis showed that Segments A and B of IBDV-JS19-14701 were derived from emerging nVarIBDV (Genotype A2dB1) and long-prevalent HLJ0504-like strains (Genotype A3B3) in China, respectively. The pathogenicity of IBDV-JS19-14701 was further evaluated via animal experiments. IBDV-JS19-14701 exhibited a similar virulence to chickens with the nVarIBDV. The identification of this reassortment event is beneficial for understanding the epidemiology of nVarIBDV and will contribute to the efficient prevention and control of IBD.

## 1. Introduction

Infectious bursal disease (IBD) is one of the most important immunosuppressive poultry infectious diseases in poultry and has caused enormous economic losses to the poultry industry worldwide. The etiological agent of IBD is infectious bursal disease virus (IBDV), which belongs to the genus Avibirnavirus in the family Birnaviridae [1]. The genome of IBDV is composed of two segments of double strand RNA, known as Segments A and B. Segment A encodes a VP5 and a polyprotein (pVP2-VP4-VP3). This polyprotein is self-cleaved by VP4 to produce VP2, VP3, and VP4 [2,3]. A hypervariable region (HVR) which spans amino acid positions from 206 to 350 of VP2 is widely used for the evolutionary analysis of IBDV [4]. Segment B encodes VP1, an RNA-dependent RNA polymerase (RdRp) that is responsible for genome replication, translation, and viral virulence of IBDV [5].

There are two serotypes of IBDV (Serotypes I and II), and only Serotype I strains are pathogenic to chickens [6,7]. Based on the pathogenicity and antigenicity, the Serotype I strain is traditionally categorized into four phenotypes; they are classic IBDV (cIBDV), variant IBDV (VarIBDV), very virulent IBDV (vvIBDV), and attenuated IBDV (aIBDV). According to an improved scheme for IBDV genotype classification [8], the cIBDV, VarIBDV, vvIBDV, and aIBDV correspond to Genotypes A1B1, A2B1 (including A2aB1, A2bB1, and A2cB1), A3B2, and A8B1, respectively. In addition, one virulent IBDV named HLJ0504-like strains (A3B3) are circulating in China [9,10,11,12,13], Pakistan [11,14], and India [15]. Recently, the epidemics caused by novel variant IBDV (nVarIBDV) were successively reported in China [16], Japan [17], Korea [18] and Malaysia [19]. The nVarIBDV strains were classified as Genotype A2dB1, which were genetically different from the early variant IBDV originally reported in America [8,18].

Genome segment reassortment is an important evolutionary feature of IBDV with a segmented genome. Herein, for the first time, we report the IBDV-JS19-14701 strain (Genotype A2dB3), a naturally occurring reassortant formed by the combination of nVarIBDV and HLJ0504-like IBDV; further, the pathogenicity of this strain was evaluated.

## 2. Materials and Methods

### 2.1. Chickens and Embryos

Specific pathogen-free (SPF) chickens and SPF embryos were supplied by the National Poultry Laboratory Animal Resource Center of the Harbin Veterinary Research Institute (HVRI), the Chinese Academy of Agricultural Sciences (CAAS). The chickens were maintained in negative-pressure-filtered air isolators.

### 2.2. Samples Collection

In November 2019, in a 30-day-old vaccinated flock of Chinese local broiler (Yao chickens) in Jiangsu province of China, the chickens grew slowly, and the growth uniformity was poor, and the atrophy of bursa was observed; in addition, the chief complaint was symptoms of immunosuppressive disorder. This flock has been immunized with one infectious bursal disease live vaccine. Five bursa tissue samples were collected from sick chickens for laboratory examination. Tissue samples were homogenized in phosphate-buffered saline (PBS; 10 mL per gram of tissue). The homogenates were frozen and thawed three times, and then centrifuged at 5000× *g* for 5 min at 4 °C, and then the supernatants were harvested and stored at −80 °C until further investigation.

### 2.3. IBDV Detection

A 1-mL aliquot of TRIzol reagent (TaKaRa, Dalian, China) was added into 0.2 mL of the bursa tissue homogenate supernatant to extract the total RNA according to the manufacturer’s instructions. The presence of IBDV in these bursa samples was detected according to a previously described RT-PCR assay targeting the HVR of the VP2 gene [8]. The RT-PCR products were detected by 1% agarose electrophoresis analysis.

### 2.4. Virus Isolation and Genome Cloning

An IBDV-positive tissue supernatant sample was randomly selected to isolate the virus; three 24-day-old SPF chickens were inoculated via the ocular and intranasal routes, as described previously [20]. At 4 days post-inoculation, the bursae were collected and processed as described in Section 2.2. To detect the purity of the isolated IBDV in the bursa samples, avian influenza virus (AIV) and Newcastle disease virus (NDV) were detected by hemagglutination assay and hemagglutination inhibition assay; Marek’s disease virus (MDV), avian leukosis virus (ALV), fowl adenovirus Serotype 4 (FAdV-4), reticuloendotheliosis virus (REV), chicken anemia virus (CAV), and mycoplasma were detected by PCR; avian metapneumovirus (aMPV) and avian reovirus (ARV) were detected by RT-PCR; bacteria was detected by LB culture for 24 h. Detection primers were list in Table S1.

The DNA fragments of viral genome of IBDV were synthesized by RT-PCR using four pairs of primers (AU/A1542L and A1421U2/AL2 for Segment A; BU/B1344L and B1344U/BL for Segment B), as described in a previous study [21]. The Segments A and B PCR products were cloned into the pMD18-T vectors (Takara, Dalian, China), respectively. The sequences of the recombinant plasmids were determined using Sanger sequencing, which was performed by Comate Biosciences Company (Changchun, China).

### 2.5. Sequence Analysis

Nucleotide alignments were performed using the MUSCLE algorithm in MEGA 6 (ASU, Phoenix, AZ, USA) [22]. Neighbor-joining trees were constructed based on the nucleotide sequence of polyprotein of Segment A and VP1 of Segment B, using the Kimura 2-parameter method in MEGA6 [23], with 1000 replicates. Genetic similarity calculation and amino acid comparations were performed using MegAlign in the DNAstar package (MegAlign, DNASTAR Inc., Madison, WI, USA). The information for the 29 representative IBDV strains used in the phylogenetic analysis was listed in Table 1. The IBDV information covering the full-length sequences of both polyprotein and VP1 are relatively not much. To further validate the genetic characteristic of IBDV-JS19-14701, based on HVR of VP2 (representative fragment of Segment A) and B-marker of VP1 (representative fragment of Segment B), the phylogenetic trees containing more strains were constructed according to the previous method [8]. The sequence information of HVR and B-marker used in the phylogenetic analysis was list in Tables S2 and S3, respectively.

### 2.6. Restriction Enzyme Digestion Assay

To exclude the possibility that the sequence information of IBDV-JS19-14701 strain came from a bursa which was co-infected with HLJ0504-like vvIBDV and nVarIBDV. The RNA of isolated IBDV-JS19-14701 sample was extracted using the method in 2.3. A 1749 bp fragment (bp 1421-bp 3170 of Segment A) containing a *Hin*d III restriction site (bp 2188 -bp 2193 of Segment A, exists in nVarIBDV but not exists in vvIBDV including HLJ0504-like strains) was amplified from the RNA sample by RT-PCR using primer set: A1421U2 (5′-GACCTCAACTCTCCCCTGAAGATTGCAGGAG-3′) and A3170L (5′-TCACTCAAGGTCCTCATCAGAGACGGT-3′). The amplicon was purified by agarose gel electrophoresis and digested with *Hin*d III restriction enzyme (NEB) overnight. The digested products were analyzed on agarose electrophoresis. In this experiment, a representative strain of nVarIBDV (SHG19 strain) [16] and a representative strain of vvIBDV (HLJ0504 strain) [11] were used as control strains.

### 2.7. Pathogenicity Evaluation

Fifteen 24-day-old SPF chickens were divided into three groups (5 chickens per group). Chickens in Groups 1 and 2 were both intraocularly and intranasally infected with 100 median tissue culture infective dose (TCID_50_) of the isolated IBDV strain and the SHG19 control strain, respectively. Chickens in Group 3 (mock control) were administered 200 μL PBS. All chickens were observed daily for clinical symptoms. At 7 days post-infection (d p. i.), all chickens were euthanized and necropsied. The weights of the body and bursa were recorded to calculate the bursa/body weight ratio (B/BW) [B/BW = bursa: body weight × 1000] [24]. Half of the bursa tissues were fixed in 4% neutral-buffered formaldehyde for histopathological examination. To detect the infection of IBDV in bursa, the immunohistochemistry (IHC) assay was performed. The bursa sections were incubated with a mouse anti-VP2 monoclonal antibody (7D4) [16] as the primary antibody and peroxidase-labeled polymer-conjugated anti-mouse immunoglobulin (Invitrogen, Waltham, MA, USA) as the secondary antibody. Immunostaining was visualized by 3,3’-diaminobenzidine tetrahydrochloride staining. Hematoxylin (Baso, Zhuhai, China) was used as a nuclear counterstain for IHC.

### 2.8. Statistical Analyses

All data were analyzed with GraphPad Prism (GraphPad Software, Inc., San Diego, CA, USA). Data were presented as the mean ± SD in all experiments. Analysis of variance (ANOVA) was used to determine the statistical significance among the groups (* *p* < 0.05).

## 3. Results

### 3.1. IBDV Detection, Isolation, and Genome Sequencing

RT-PCR targeting the HVR of VP2 confirmed that all five bursa samples were positive for IBDV. One IBDV strain was successfully isolated form a randomly selected bursa suspension and designated as IBDV-JS19-1470 strain. The isolated IBDV-JS19-1470 strain is pure and is free of other pathogens including AIV, NDV, MDV, ALV, FAdV-4, REV, CAV, aMPV, ARV, mycoplasma, and bacteria.

The final consensus genomic sequence of IBDV-JS19-14701 was determined by Sanger sequencing. Sequencing, which showed that the Segment A of IBDV-JS19-14701 (3260 nucleotides, nt) consisted of a 5’-non-coding region (NCR, 84 nt), a 3’-NCR (91 nt), a small VP5-encoding ORF (450 nt), and a large ORF (3039 nt) encoding the polyprotein (pVP2-VP4-VP3). Segment B was composed of a VP1-coding ORF (2637 nt) flanked by a 5’-NCR (111 nt) and a 3’-NCR (79 nt). The genome sequences of IBDV-JS19-14701 were submitted to GenBank (Accession Numbers: Segment A-MW700332; Segment B-MW700333).

### 3.2. Sequence Analysis of IBDV-JS19-14701 Genome

**Figure 1:**
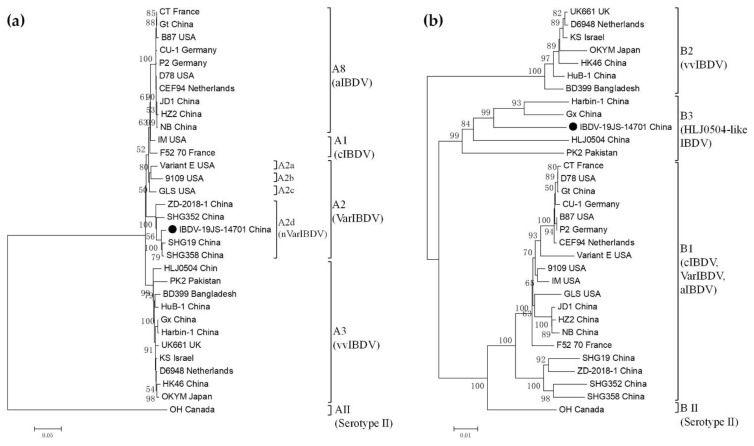
Phylogenetic analysis of the nucleotide sequences encoding the polyproteins (**a**) and VP1 (**b**). The trees were generated by the neighbor-joining method with MEGA6 software. Trees were drawn to scale, with branch lengths measured in the number of substitutions per site. Only branches supported by a bootstrap value above 50% were displayed. The genogroup and corresponding phenotype for each branch was marked. The segment-reassortant strain detected in this study (IBDV-JS19-14701) was highlighted with a solid circle.

The phylogenetic trees based on the nucleotide sequences of the polyprotein in Segment A showed that Serotype I strain contained four previously described genogroups: A1 (cIBDV), A2 (VarIBDV), A3 (vvIBDV), and A8 (aIBDV) [8]. Genogroup A2 included lineages A2a/b/c (early variant IBDV) and A2d (nVarIBDV) [8,18]. The IBDV-JS19-14701 strain was clustered with SHG19 [16], which is a representative strain of nVarIBDV (A2d) (Figure 1a). Based on the nucleotide sequences of VP1 in Segment B, the Serotype I representative strains were divided into three genogroups, B1, B2, and B3, and IBDV-JS19-14701 belonged to the Genogroup B3 (HLJ0504-like strains) but not Genogroup B1 (including SHG19-like strain) (Figure 1b). In addition, the phylogenetic trees based on HVR of VP2 and B-marker of VP1 also showed that the IBDV-JS19-14701 strain contained nVarIBDV Segment A (Genogroup A2d) and HLJ0504-like Segment B (Genogroup B3) (Figure S1).

The nucleic acid identity and amino acid comparation further confirmed the different origins of the two genome segments of the IBDV-JS19-14701 strain. In Segment A, genetic distances derived from a comparison of the nucleic acid sequences revealed that the polyprotein of IBDV-JS19-14701 shared a higher similarity (96.3–98.8%) with nVarIBDV strains (Genogroup A2d) than with other representative strains (93.2–95.0%). The characteristic amino acid residues in Segment A of IBDV-JS19-14701 exhibited a SHG19-like profile, except for one specific alteration in VP5 (78 L) (Table 2). In Segment B, the nucleic acid sequence of IBDV-JS19-14701 VP1 exhibited 92.7–95.3% identity with HLJ0504-like strains (Genogroup B3), which is higher than its identity with the nucleic acid sequence of the VP1 of novel variant IBDV strains (90.2–90.3%). All 15 characteristic amino acids in IBDV-JS19-14701 VP1 were identical to those in the representative strain HLJ0504 among the HLJ0504-like strains (Table 2).

**Figure 2:**
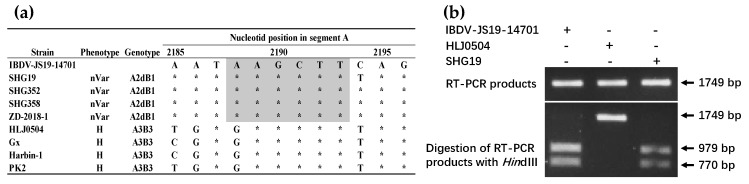
Identification of co-infection by restriction enzyme digestion analysis. (**a**) A part of the nucleotide alignment report showing the presence or absence of the *Hin*d III restriction enzyme site (bp 2188–bp 2193) in Segment A. An asterisk indicates a residue identical to the IBDV-JS19-14701 strain. The shaded area indicates the presence of a *Hin*d III restriction site; (**b**) total RNA of IBDV-JS19-14701, SHG19, or HLJ0504 was extracted and subjected to RT-PCR, respectively. The RT-PCR products (bp 1421–bp 3170 of Segment A, 1749 bp) were then subjected to *Hin*d III digestion.

To further validate that the sequence of IBDV-JS19-14701 strain is indeed from a single viral strain but not the result of co-infection of two strains of SHG19-like and HLJ0504-like strain, we performed a restriction enzyme digestion assay targeting the *Hin*d III restriction site which exist in SHG19-like strain but not in HLJ0504-like strain (Figure 2a). The result showed that the 1749 bp RT-PCR product of IBDV-JS19-14701 was completely cleaved by the *Hin*d III restriction enzyme, and the SHG19 strain had the same restriction enzyme digestion profile (Figure 2b). However, the RT-PCR product of HLJ0504 was not cleaved by the *Hin*d III restriction enzyme (Figure 2b).

These results suggested that IBDV-JS19-14701 strain was a segment reassortment IBDV with a nVarIBDV Segment A (Genogroup A2d) and a HLJ0504-like Segment B (Genogroup B3). According to the new classification scheme [8], nVarIBDV and HLJ0504-like IBDV was corresponded to Genotypes A2dB1 and A3B3, respectively. Therefore, the genotype of the segment reassortment strain IBDV-JS19-14701 can be defined as A2dB3.

### 3.3. Pathogenicity Experiment

**Figure 3:**
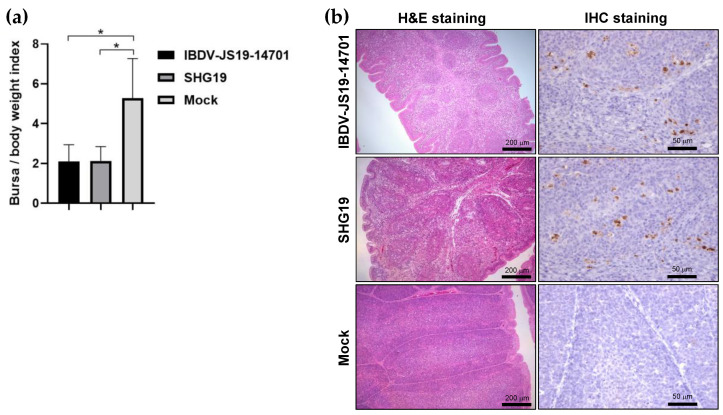
Pathogenicity evaluation of the reassortant strain IBDV-JS19-14701. (**a**) B/BW ratio at seven days post-infection (* *p* < 0.05); (**b**) Representative hematoxylin and eosin (H&E) staining (scale bars, 200 μm) and immunohistochemistry (IHC) staining (scale bars, 50 μm) of IBDV VP2 protein in the bursal tissues harvested at seven days post-infection.

To evaluate the pathogenicity of IBDV-19JS-14701 strain, an animal experiment was performed. During the investigation period, no clinical symptoms and mortality were observed in either the IBDV-JS19-14701 infection group or the nVarIBDV SHG19 control group. The results of the autopsy, performed at 7 d. p. i., showed that IBDV-JS19-14701 induced severe atrophy of the bursa (B/BW = 2.10 ± 0.84), which was significantly different from that in the mock group (B/BW = 5.28 ± 1.99) (*p* < 0.05). Similar atrophy of the bursa (B/BW = 2.13 ± 0.72) was observed in the SHG19 control group (Figure 3a). Histopathological examination further confirmed that both IBDV-JS19-14701 and SHG19 induced severe damage to the bursa including atrophy of the follicles, lymphocyte depletion, and hyperplasia of fibrous tissue (Figure 3b). No lesions appeared in any of the mock chickens. IHC results showed obviously positive signals in the bursae of IBDV-JS19-14701-infection group and SHG19-infection group. No positive signal was observed in the bursae of mock group (Figure 3b).

## 4. Discussion

Reassortment is an important mechanism in the genetic evolution of IBDV, which has a two-segmented genome. The random exchange of genome segments between viruses in different genotypes has led to the emergence of new IBDV genotypes [25]. It has been reported that the reassortment of Segment A from very virulent strain (Genotype A3B2) and Segment B from attenuated strain (A8B1) resulted in the new segment-reassortant IBDV of genotypes of A3B1, which has been identified in China [21,26], India [27], Korea [28], Poland [29], Venezuela [30], Ethiopia [31], Nigeria [32], and Zambia [33]. Meanwhile, Segment A of attenuated strain (A8B1) and Segment B of very virulent strain (A3B2) were also recombined to generate a segment-reassortant IBDV with Genotype A8B2 [25,34]. In addition, in the USA and France, the segment-reassortant IBDVs of Genotype A3/BII (Segment A from vvIBDV and Segment B from the Serotype II strain) have been observed [35,36,37]. Our present study reported a novel IBDV reassortant of Genotype A2dB3 carrying Segment A of nVarIBDV (A2dB8) and Segment B of HLJ0504-like strain (A3B3). Further studies should be conducted to determine the origin of the IBDV-JS19-14701 strain and the mechanisms underlying the reassortment of segmented viruses.

The HLJ0504-like IBDVs (A3B3) are the main prevalent strains with high pathogenicity, which have been threatening the poultry farming in China for nearly 30 years [9,10,11,12]. Such type of IBDVs have also been circulating in the neighboring countries including Pakistan [11,14], India [15], Bangladesh [38], Thailand, Vietnam [39], and Korea [18], and were also reported in Venezuela [30]. Recently, the widespread of nVarIBDV (A2dB1) was discovered in China [16,40] and also reported in Japan [17], Korea [18], and Malaysia [19] in succession. The damage caused by nVarIBDV to the poultry industry has been well documented that nVarIBDV can cause severe bursal lesions and immunosuppression [16,41,42]. The co-existence of emerging nVarIBDV and long-prevalent HLJ0504-like strains provided the opportunity to generate reassortants through complex mechanisms, including the influence of the host, selection pressure, and environmental factors. It has been reported some available vaccines cannot protect against nVarIBDV well [42]. The flock in this study has been immunized with IBD live vaccine. Obviously, the reassortant IBDV-JS19-14701 with Segment A of nVarIBDV escaped the immune-protection of vaccine to some extent.

The pathogenicity of IBDV is determined by both segments of the genome [43], and segment reassortment can influence the virulence of IBDV. One study has confirmed that a naturally occurring reassortant strain with Segment A from vvIBDV and Segment B from attenuated IBDV has lower pathogenicity than that of vvIBDV [21]. Another study also showed that the segment reassortment between attenuated strains and vvIBDVs increased the pathogenicity of attenuated strain [44]. It has been identified that nVarIBDV is the main cause of the subclinical infection of IBD, which cannot kill chickens directly but acutely damages bursa resulting in severe immunosuppression and growth retardation [16,42,45,46]. The HLJ0504-like strain is a virulent IBDV with a high fatality rate [11]. Whether the replacement of Segment B of HLJ0504-like strain increased the pathogenicity of nVarIBDV is an issue worthy of attention. In the animal experiments, the reassortant IBDV-JS19-14701 showed similar pathogenicity to chickens as the representative nVarIBDV of SHG19 which could cause severe bursal damages. In this study, we just compared the pathogenicity between IBDV-JS19-14701 and SHG19 strains, and the detailed pathogenicity comparisons among three kinds of IBDV (IBDV-JS19-14701, SHG19, and HLJ0504) and its molecular mechanism needs further studies. The recombination between nVarIBDV and long-prevalent HLJ0504-like strain further hints the complexity of evolution and epidemic and the severity of prevention and control of IBD.

## 5. Conclusions

In conclusion, it was firstly identified of the naturally genome-reassortant IBDV with a nVarIBDV Segment A and a HLJ0504-like Segment B. The segment-reassortment of IBDV posed additional challenges to the healthy development of poultry farming.

## Figures and Tables

**Table 1:** IBDV representative strains used in the sequence alignment and phylogenetic analysis.

Strains	Phenotype ^1^	Genotype	GenBank Accession No.		Strains	Phenotype ^1^	Genotype	GenBank Accession No.	
			Segment A	Segment B				Segment A	Segment B
IM	C	A1B1	AY029166	AY029165	Harbin-1	H	A3B3	EF517528	EF517529
F52/70	C	A1B1	HG974565	HG974566	Gx	H	A3B3	AY444873	AY705393
Variant E	Var	A2aB1	AF133904	AF133905	PK2	H	A3B3	AY368653	AY368654
9109	Var	A2bB1	AY462027	AY459321	HLJ0504	H	A3B3	AF133904	AF133905
GLS	Var	A2cB1	AY368653	AY368654	CU-1	A	A8B1	X16107	AF362775
SHG19	nVar	A2dB1	MN393076	MN393077	JD1	A	A8B1	AF321055	AY103464
SHG352	nVar	A2dB1	MT179720	MT179722	B87	A	A8B1	DQ906921	DQ906922
SHG358	nVar	A2dB1	MT179721	MT179723	Gt	A	A8B1	DQ403248	DQ403249
ZD-2018-1	nVar	A2dB1	MN485882	MN485883	CT	A	A8B1	AJ310185	AJ310186
OKYM	VV	A3B2	D49706	D49707	P2	A	A8B1	X84034	X84035
HK46	VV	A3B2	AF092943	AF092944	NB	A	A8B1	AY319768	AY654284
D6948	VV	A3B2	AF240686	AF240687	CEF-94	A	A8B1	AF194428	AF194429
KS	VV	A3B2	DQ927042	DQ927043	HZ2	A	A8B1	AF321054	AF493979
UK661	VV	A3B2	NC-004178	NC-004179	D78	A	A8B1	AF499929	AF499930
HuB-1	VV	A3B2	KF569805	GQ449693	OH	Serotype II	AIIBII	U30818	U30819
BD399	VV	A3B2	AF362776	AF362770					

^1^ C, classic strain; Var, variant strain; nVar, novel variant strain; VV, very virulent strain; H: HLJ0504-like strain; A, attenuated strain.

**Table 2:** Comparison of the characteristic amino acid residues between the novel variant strain (nVar) and HLJ0504-like IBDV (H).

		Segment A																												Segment B														
		VP5			VP2																		VP4					VP3		VP1														
Strain	Phenotype	49	78	129	213	221	222	242	249	252	253	254	256	279	284	286	294	299	318	323	330	451	541	680	685	715	751	981	1005	4	61	145	146	147	242	287	390	508	511	546	562	646	687	**695**
IBDV-19JS-14701		G	F	P	N	K	T	V	K	I	Q	N	V	N	A	I	L	S	D	E	S	L	I	C	K	P	H	P	A	V	I	T	E	G	D	A	L	K	S	P	S	S	P	K
SHG19	nVar	*	L	*	*	*	*	*	*	*	*	*	*	*	*	*	*	*	*	*	*	*	*	*	*	*	*	*	*	I	V	N	*	D	*	T	*	*	R	*	*	G	*	*
SHG352	nVar	*	L	*	*	*	*	*	*	*	*	*	*	*	*	*	*	*	*	*	*	I	*	*	*	*	*	*	*	I	V	N	*	D	*	T	*	*	R	*	*	G	*	*
SHG358	nVar	*	L	*	*	*	*	*	*	*	*	*	*	*	*	*	*	*	*	*	*	*	*	*	*	*	*	*	*	I	V	N	V	V	*	T	*	*	R	*	*	G	S	*
ZD-2018-1	nVar	*	L	*	D	*	*	*	*	*	*	*	*	*	*	*	*	*	*	*	*	I	*	*	*	*	*	*	*	I	V	S	*	D	*	T	*	*	R	*	*	G	S	*
HLJ0504	H	R	*	*	D	Q	A	I	Q	V	*	G	I	D	*	T	I	*	G	D	*	*	*	Y	N	S	D	*	*	*	*	*	*	*	*	*	*	*	*	*	*	*	*	*
Gx	H	R	*	*	D	Q	A	I	Q	V	*	G	I	D	*	T	I	*	G	D	*	*	*	Y	N	S	D	*	*	*	*	*	*	*	*	*	*	*	*	*	*	*	*	*
Harbin-1	H	R	*	*	D	Q	A	I	Q	V	*	G	I	D	*	T	I	*	G	D	*	*	*	Y	N	S	D	*	*	*	*	*	*	*	*	*	*	*	*	S	*	*	*	*
PK2	H	R	*	S	D	Q	A	I	Q	V	*	G	I	D	*	T	I	*	G	D	*	*	*	Y	N	S	D	*	*	*	*	S	*	*	*	*	*	*	*	*	*	*	S	*

An asterisk indicates a residue identical to the IBDV-JS19-14701 strain.

## Data Availability

Data can be requested by writing to the author.

## Supplementary Materials

The following are available online at https://www.mdpi.com/article/10.3390/v13091682/s1, Figure S1: phylogenetic analysis of the nucleotide sequences encoding the HVR of VP2 (a) and B-marker of VP1 (b), Table S1: The primers for detecting other pathogens, Table S2: The HVR sequence information of IBDV representative strains, Table S3: The B-marker sequence information of IBDV representative strains.
