# Supplementary material for: Identification and Pathogenicity Evaluation of a Novel Reassortant Infectious Bursal Disease Virus (Genotype A2dB3)
Source: Viruses. 2021 Aug 25;13(9):1682. doi: 10.3390/v13091682 (PMC8472943; doi:10.3390/v13091682)
Supplement: Supplementary file 1 [file viruses-13-01682-s001.zip › viruses-1302792-supplementary/Supplementary Figure S1.pdf]

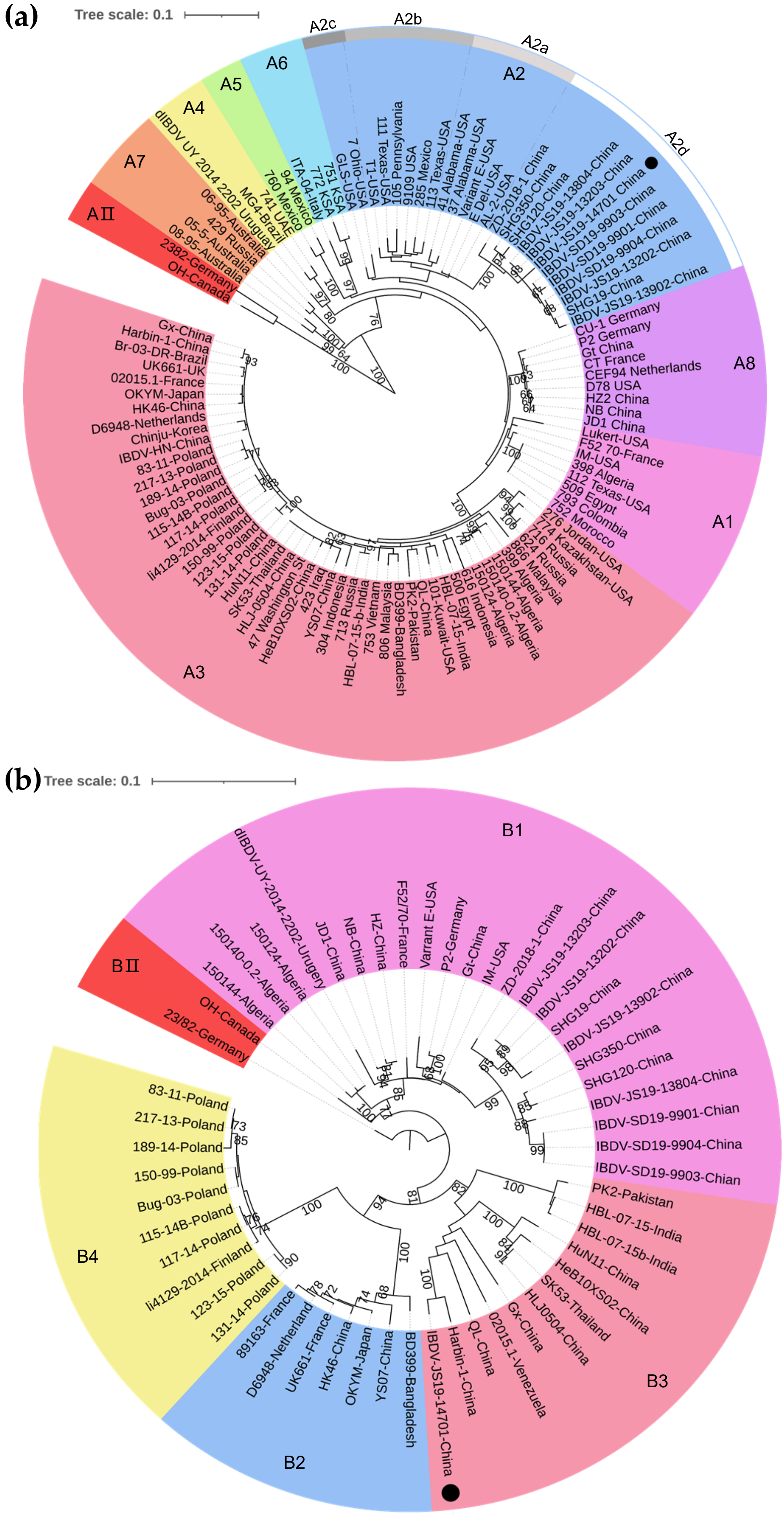

Figure S1. Phylogenetic analysis of the nucleotide sequences encoding the HVR of VP2 (a) and B-marker of VP1 (b). The trees were generated by the Maximum-likelihood method with MEGA6 software. Trees were drawn to scale, with branch lengths measured in the number of substitutions per site. Only branches supported by a bootstrap value above 60% were displayed. The genogroup for each branch was marked. The segment-reassortant strain detected in this study (IBDV-JS19-14701) was highlighted with a solid circle.
