# Supplementary material for: Identification and Pathogenicity Evaluation of a Novel Reassortant Infectious Bursal Disease Virus (Genotype A2dB3)
Source: Viruses. 2021 Aug 25;13(9):1682. doi: 10.3390/v13091682 (PMC8472943; doi:10.3390/v13091682)
Supplement: Supplementary file 1 [file viruses-13-01682-s001.zip › viruses-1302792-supplementary/Supplementary Tables.pdf]

Table S1 The primers for detecting other pathogens.

Pathogens	Primers	Sequence 5′-3′
MDV	upstream	TGCGATGAAAGTCTATGGAGG
	downstream	GAGAATCCCTATGAGAAAGCGC
ALV-A	upstream	CGGAGAAGACACCCTTGCT
	downstream	GCATTGCCACAGCGGTACTG
ALV-B	upstream	CGGAGAAGACACCCTTGCT
	downstream	GTAGACACCAGCCGGACTATC
ALV-J	upstream	CGGAGAAGACACCCTTGCT
	downstream	CGAACCAAAGGTAACACACG
ALV-K	upstream	CGGAGAAGACACCCTTGCT
	downstream	TCTGAAAGCTTGTCAATGCTCCG
FAdV-4	upstream	GCCACCGGAAGCTACTTTGA
	downstream	TTGTGATCCATGGGCATGA
REV	upstream	GCCTTAGCCGCCATTGTA
	downstream	CCAGCCTACACCACGAACA
CAV	upstream	CGGTCGGCAGTAGGTAT
	downstream	CGATACCGCTGTCTCCT
aMPV	upstream	ATGCAAGCTTATGGAGCTGG
	downstream	AACACTTGCAAAGTTGGG
ARV	upstream	TAAGCACAATGCAATGGCTCCGCC
	downstream	TGAGACCCGCCATCCCAATGAACT
mycoplasma	upstream	AAAGCAAACTATGTGCCAGCAG
	downstream	CACTCGTAAGAGGCATGATGA

Table S2 The HVR sequence information of IBDV representative strains

Genogroup		Feature[1]	IBDV strain	GenBank No.	Genogroup	Feature[1]	IBDV strain	GenBank No.
A1		C	F52/70 France	HG974565			83/11 Poland	KX759603
			IM USA	AY029166			217/13 Poland	KX759610
			Lukert USA	AY918948			115/14B Poland	KX759606
			112_Texas USA	MF142505			117/14 Poland	KX759607
			398_Algeria	MF142527			131/14 Poland	KX759608
			509_Egypt	MF142543			189/14 Poland	KX759611
			793_Colombia	MF142581			123/15 Poland	KX759614
			752_Morocco	MF142572			li4129/2014 Finland	MG739298
A2	a[2]	Var	AL-2 USA	JF736011			716_Russia	MF142563
			E Del USA	DD187400			HeB10XS02 China	KF569801
			37_Alabama USA	MF142526			QL China	JX682709
			41_Alabama USA	MF142532			HuN11 China	LM651367
			Variant E USA	AF133904			SK53 Thailand	KJ198843
	b[2]		T1 USA	AF281238			150140/0.2 Algeria	MF969119
			111_Texas USA	MF142504			150144 Algeria	KY555598
			105_Pennsylvania USA	MF142503			150124 Algeria	KY555572
			9109 USA	AY462027			276_Jordan	MF142517
			113_Texas USA	MF142506			500_Egypt	MF142542
			285_Mexico	MF142520			304_Indonesia	MF142521
	c[2]		GLS USA	AY368653			753_Vietnam	MF142573
			7_Ohio USA	MF142583			713_Russia	MF142562
	d[2]		SHG19 Chian	MH879092			423_Iraq	MF142535
			SHG120 China	MH879110			47_Washington_St USA	MF142539
			SHG350 China	MH879129			806_Malaysia	MF142584
			IBDV-SD19-9901 Chian	MT087547			866_Malaysia	MF142587
			IBDV-SD19-9903 Chian	MT087548			399_Algeria	MF142528
			IBDV-SD19-9904 China	MT087549			616_Indonesia	MF142551
			IBDV-JS19-13202 China	MT087550			PK2 Pakistan	MF996499
			IBDV-JS19-13203 China	MT087551	A4	dIBDV	UY/2014/2202 Uruguay	KT336459
			IBDV-JS19-13804 China	MT087552			741_UAE	MF142569
			IBDV-JS19-13902 China	MT087553			MG4 Brazil	JN982252
			ZD-2018-1	MN485882	A5	V/C Recomb	760_Mexico	MF142575
A3		VV	UK661 France	NC_004178			94_Mexico	MF142589
			OKYM Japan	D49706	A6	ITA	772_KSA	MF142577
			BD339 Bangladesh	AF362776			751_KSA	MF142571
			HLJ0504 China	GQ451330			ITA-04 Italy	JN852988
			Gx China	AY444873	A7	Australia	08/95 Australia	AF148081
			HK46 China	AF092943			06/95 Australia	AF148080
			YS07 China	FJ695138			05-5 Australia	AF381011
			Br/03/DR Brazil	EU835860			429_Russia	MF142536
			IBDV-HN China	KT884486	A8	A	D78 USA	AF499929
			Chinju Korea	AF508176			CT France	AJ310185
			Harbin-1 China	EF517528			P2 China	X84034
			D6948 Netherlands	AF240686			CEF94 Netherlands	AF133904
			02015.1 Venezuela	AJ878909			JD1 China	AF321055
			HBL-07-15 India	KT630853			HZ2 China	AF321054
			HBL-07-15-b India	KT630855			NB China	AY319768
			101_Kuwait	MF142502			CU-1 Germany	X16107
			624_Russia	MF142552			Gt China	DQ403248
			774_Kazakhstan	MF142578	BII	serotype II	23/82 Germany	AF362773
			150/99 Poland	KX759601			OH Canada	U30818
			Bug/03 Poland	KX759602				

[1] C, classic strain; Var, variant strain; VV, very virulent IBDV; dIBDV, distinct IBDV; V/C Recomb, Variant / Classic recombinant strain; ITA, ITA-like strains; A, attenuated strain; nVar, novel variant strains; Serotype II, Serotype II IBDV. [2] Lineages are defined in genogroup A2 using lowercase letters.

Table S3 The B-marker sequence information of IBDV representative strains

Genogroup	Feature[1]	IBDV strain	GenBank No.	Genogroup	Feature[1]	IBDV strain	GenBank No.
B1	C	F52/70 France	HG974566			OKYM Japan	D49707
		IM USA	AY029165			BD339 Bangladesh	AF362770
	Var	Variant E USA	AF133905			89163 France	HG974564
	A	HZ2 China	AF493979	B3	H	HLJ0504 China	GQ451331
		NB China	AY654284			Gx China	AY705393
		JD1 China	AY103464			Harbin-1 China	EF517529
		P2 Germany	X84035			PK2 Pakistan	MF996500
		Gt China	DQ403249			02015.1 Venezuela	AJ880090
	dIBDV	UY/2014/2202 Uruguay	KT336458			HeB10XS02 China	KF569802
	Algeria	150124 Algeria	KY555573			QL China	JX682709
		150140/0.2 Algeria	MF969121			HuN11 China	LM651367
		150144 Algeria	KY555599			HBL-07-15 India	KT630845
	nVar	SHG19 China	MH879045			HBL-07-15b India	KT630847
		SHG120 China	MH879063			SK53 Korea	KJ198843
		SHG350 China	MH879081	B4	Trans	150/99 Poland	KX759555
		IBDV-SD19-9901 China	MT087554			Bug/03 Poland	KX759556
		IBDV-SD19-9903 China	MT087555			li4129/2014 Poland	MG739299
		IBDV-SD19-9904 China	MT087556			83/11 Poland	KX759557
		IBDV-JS19-13202 China	MT087557			217/13 Poland	KX759559
		IBDV-JS19-13203 China	MT087558			115/14B Poland	KX759561
		IBDV-JS19-13804 China	MT087559			117/14 Poland	KX759562
		IBDV-JS19-13902 China	MT087560			131/14 Poland	KX759563
		ZD-2018-1	MN485883			189/14 Poland	KX759565
B2	VV	UK661 France	NC-004179			123/15 Poland	KX759568
		YS70 China	FJ695139	BII	Serotype II	23/82 Germany	AF362774
		D6948 Netherlands	AF240687			OH Canada	U30819
		HK46 China	AF092944				

[1] C, classic strain; Var, variant strain; A, attenuated strain; dIBDV, distinct IBDV; Algeria, the recombinant strain isolated in Algeria; H, HLJ0504-like strain; VV, very virulent IBDV; nVar, novel variant strains; Trans, transitional-lineage IBDV; Serotype II, serotype II IBDV.
